# Pro-Inflammatory Cytokines but Not Endotoxin-Related Parameters Associate with Disease Severity in Patients with NAFLD

**DOI:** 10.1371/journal.pone.0166048

**Published:** 2016-12-19

**Authors:** Johannie du Plessis, Hannelie Korf, Jos van Pelt, Petra Windmolders, Ingrid Vander Elst, An Verrijken, Guy Hubens, Luc Van Gaal, David Cassiman, Frederik Nevens, Sven Francque, Schalk van der Merwe

**Affiliations:** 1 Laboratory of Hepatology, Department of Clinical and Experimental Medicine, KU Leuven, Leuven, Belgium; 2 Translational Research Center for Gastrointestinal Disorders (TARGID), Department of Clinical and Experimental Medicine, KU Leuven, Leuven, Belgium; 3 Department of Endocrinology, Diabetology and Metabolism, Antwerp University Hospital, University of Antwerp, Antwerp, Belgium; 4 Department of Abdominal Surgery, Antwerp University Hospital, University of Antwerp, Antwerp, Belgium; 5 Department of Gastroenterology and Hepatology, Antwerp University Hospital, University of Antwerp, Antwerp, Belgium; 6 Department of Internal Medicine, Division of Liver and biliopancreatic disorders, KU Leuven, Leuven, Belgium; Medizinische Fakultat der RWTH Aachen, GERMANY

## Abstract

Intestinal dysbiosis and elevated lipopolysaccharides (LPS) levels have been implicated in the development of obesity, insulin resistance and non-alcoholic steatohepatitis (NASH). In order to determine if LPS levels are elevated in patients with NASH compared to patients with non-alcoholic fatty liver (NAFL) and, if elevated LPS levels correlated with histological severity of non-alcoholic fatty liver disease (NAFLD) we compared LPS, markers of LPS bioactivity and pro-inflammatory cytokines/chemokines in patients undergoing bariatric surgery. At the time of surgery a liver biopsy was taken allowing the stratification into well-delineated subgroups including: No NAFL/NAFL; NASH; NASH with fibrosis and NASH cirrhotics, using the NAFLD Activity Score (NAS). Anthropometric data and plasma were collected for assessment of LPS, lipopolysaccharide binding protein (LBP), soluble CD14 (sCD14), intestinal-type fatty acid binding protein (iFABP), Toll-like receptors 2 and 4 (TLR2, 4) and a panel of cytokines/chemokines. Similar analysis was performed on plasma from a cohort of healthy controls. Our data indicate elevated levels of LPS, LBP, sCD14, iFABP and TLR2,4 in obese patients compared to healthy controls, however, these parameters remained unaltered within patients with limited liver disease (NAFL) compared to NASH/NASH with fibrosis subgroups. Hierarchic cluster analysis using endotoxin-related parameters failed to discriminate between lean controls, NAFLD. While similar cluster analysis implementing inflammation-related parameters clearly distinguished lean controls, NALFD subgroups and NASH cirrhotics. In addition, LPS levels was not associated with disease severity while TNFα, IL8, and CCL3 featured a clear correlation with transaminase levels and the histological severity of NALFD. In conclusion our data indicate a stronger correlation for circulating inflammatory- rather than endotoxin-related parameters in progression of NAFLD and highlights the need for additional larger studies in unravelling further mechanistic insights.

## Introduction

Non-alcoholic fatty liver disease (NAFLD), the hepatic manifestation of the metabolic syndrome [1–3], is characterized by the development of simple steatosis or non-alcoholic fatty liver (NAFL), a condition that runs a benign course. However, in approximately 20% of the patients the disease may progress to inflammation and hepatocyte degeneration referred to as non-alcoholic steatohepatitis (NASH) due to mechanisms incompletely understood. NASH is a very serious condition which predisposes individuals to progressive fibrosis, cirrhosis and hepatocellular carcinoma [4].

Important work over the last decade has shed light on the intricate cross talk between the gut and intestinal microbiota in obesity and how changes in microbiota composition and diversity may influence NAFLD pathogenesis in animal models [5,6]. These studies showed that altered bacterial flora in obese mice harvested energy more efficiently and that weight gain could be transferred from obese to lean mice [7]. When obese mice were kept with non-obese littermates the latter developed obesity, insulin resistance and steatosis. This important observation linked obesity to the transmission of intestinal bacteria, suggesting that bacterial products play an important role in the development obesity-induced metabolic alterations [7]. Changes in gut microbiota in obesity have also been linked to an increase in gut permeability and systemic inflammation [8–12].

LPS a constituent of the cell wall of Gram-negative bacteria is a potent inflammatory trigger signaling through the TLR4/NF-κB signaling pathway [13]. LPS promotes the development of obesity and steatosis in animals models even in the absence of high-fat diet [8,14,15]. Furthermore hepatic inflammation, lipid peroxidation and insulin resistance were markedly reduced in mice deficient for TLR4 suggesting a role for LPS and TLR4 in steatohepatitis mouse models [16]. This led to the concept of “metabolic endotoxaemia” where LPS could be linked to the development of weight gain, insulin resistance and steatosis in mice. Studies in human subjects have also shown that the composition of the microbiome in NASH is altered and LPS levels are elevated compared to lean individuals [17,18]. The consequences of an altered microbiome, be it through the more efficient extraction of energy, increased permeability or translocation of bacterial products may thus also contribute to the development of NASH in human beings. However in humans NASH develops in only 20% of obese subjects which suggests that the pathogenesis of NAFLD may be different in human beings compared to animal models.

In this study we specifically assessed the role of various markers of endotoxemia in well-characterized bariatric patients stratified in distinct clinically relevant histological subgroups of NAFLD. We provide evidence that markers of endotoxemia are not different when comparing obese patients with normal liver histology, NAFL or NASH. Instead, we show that increased levels of the cytokines/chemokines IL8, TNFα and CCL3 correlated with markers of NAFLD disease severity including liver inflammation and fibrosis scores.

## Methods

### Study population

A prospective cohort study was performed in severely obese Caucasian patients undergoing bariatric surgery at the university hospital UZ Antwerp between January 2007 and October 2012. This patient population has been recently described [19,20]. Anthropometric data was obtained and plasma collected before bariatric surgery [19,20]. At surgery a liver biopsy (16G Trucut needle biopsy) was performed in all patients. Patients were excluded if they consumed more than 20 g of ethanol per day or if any other etiology of chronic liver disease became apparent during biochemical or serological testing or, upon pathological evaluation of the liver specimen. We specifically excluded diabetic patients in this study to ensure that the pro-inflammatory state associated with diabetes does not influence the findings. Patients were followed by a multi-disciplinary team of specialists, a prerequisite for reimbursement in Belgium. Medication use, dietary habits and smoking status were prospectively recorded in all patients. In addition, healthy blood donors as well as decompensated NASH cirrhotics at the time of evaluation for liver transplantation were included. The study was approved by the ethics committees of the Antwerp University hospital and University of Leuven and each participant gave written informed consent and all methods were carried out in accordance to these guidelines.

**Fig 1:**
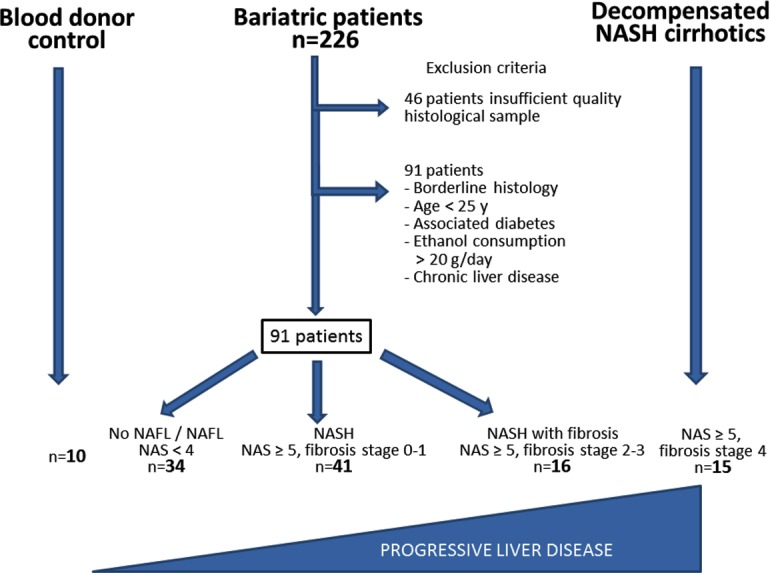
A schematic representation of the study design

Patients that met the inclusion criteria and gave informed consent were subjected to bariatric surgery. Forty six patients were excluded because of insufficient quality of the liver biopsy. Ninety one patients were excluded because of a borderline histology for NASH, or age below 25 years or associated diabetes. Finally, a total of 91 patients were included in this study as well as 10 blood donor controls and 15 decompensated NASH cirrhotics. Patients with NASH cirrhosis had no evidence of bacterial infections at the time of inclusion (Fig 1).

### Liver histology

Histological scoring was performed by an expert pathologist blinded to all clinical information, according to the NASH-Clinical Research Network Scoring System [21]. Severity of disease was assessed using the NAS (NAFLD Activity Score) as the unweighted sum of scores of steatosis, hepatocyte ballooning and lobular inflammation where NASH was defined as necessitating the simultaneous presence of any degree of steatosis, lobular inflammation and ballooning (S1 Table) [21]. A liver biopsy < 2cm and/or portal tracts <5 or borderline features of NASH (NAS of 3–4) were considerate as inadequate and excluded for further analysis. According to the liver histology 3 subgroups were identified:

Subgroup 1 (No NAFL/NAFL): included patients selected for bariatric surgery with <5% steatosis, by definition no NAFLD. NAS score for all patients in this group is 0 and patients with simple steatosis or Non-alcoholic Fatty Liver (NAFL = NAFLD but no NASH).

Subgroup 2 (NASH): NASH without advanced fibrosis (fibrosis score 0–2).

Subgroup 3 (NASH with fibrosis): NASH with advanced fibrosis (fibrosis score 3), but no cirrhosis.

### Sample collection

Blood was aseptically collected in the fasting state prior to surgery from a peripheral vein, centrifuged and stored at -80°C until further analysis.

### Biochemical analysis

Standard blood investigations were done including liver function tests, lipid profile, glucose levels. In addition, fasting insulin was determined and the HOMA-IR calculated according to the following formula ((insulin [mU/L] x glucose [mmol/L])/22.5). Other liver pathology was excluded by appropriate biochemical and serological tests.

### Plasma biomarkers

#### Plasma endotoxin (LPS) levels

Samples were diluted 1:3 with LAL reagent water and heat inactivated for 30min at 65°C. All samples were analyzed in duplicate according to the manufacturer’s instructions, using the Limulus Amoebocyte Lysate (LAL) assay QCL-1000 (Lonza, Valais Switzerland).

#### Markers of LPS bioactivity and enterocyte damage

Lipopolysaccharide binding protein (LBP), Soluble CD14 (sCD14) and Intestinal-type fatty acid binding protein (iFABP) were assessed using commercially available enzyme-linked immunosorbent assays (HyCult Biotechnologies B.V, Uden, Netherlands) and samples were tested in duplicate in 96 well plates.

#### Toll-like receptor measurements

Plasma levels of Toll-like receptor 2, 4 (TLR2, 4) were determined in duplicate according to manufactures instructions using enzyme-linked immunosorbent assays (SEA663Hu, SEA753Hu: Cloud-Clone Corporation, Houston, Texas, USA).

#### Cytokine measurements

Meso Scale Discovery V-plex assays (Rockville, Maryland, USA) were used to determine plasma cytokine (IL10, IL1β, IL6, IL8, TNFα,) and chemokine (monocyte chemotactic protein 1 (MCP1), monocyte chemotactic protein 4 (MCP4), macrophage derived chemokine (MDC), macrophage inflammatory protein 1 alpha (MIP-1α/CCL3)) levels. All measurements were performed in duplicate according to the specifications of the provider (S2 Table).

#### Statistical Analysis

All data are presented as either mean with standard deviation or median with interquartile ranges. Statistical analysis for group comparisons was performed using Kruskal-Wallis or Mann-Whitney-Wilcoxon rank sum tests where appropriate with *post-hoc* correction for multiple comparisons. The Spearman correlation test was used to determine associations between variables. All analysis was performed with Sigma Stat 3.5 (Jandel Scientific Software, San Rafael, CA) and p<0.05 was considered statistically significant. The software package PermutMatrix was used for hierarchic clustering and seriation analysis [22].

## Results

### Clinical characteristics

Table 1 summarizes the anthropometric, clinical and biochemical data of all the patients. Clinical and biochemical data of the cirrhosis patients can be found in S3 Table.

**Table 1:** Clinical and biochemical characteristics, plasma biomarkers, proinflammatory cytokines and chemokines measured in sub groups of patients with different stages of NAFLD.

	No NAFL and NAFL (n = 34)	NASH (n = 41)	NASH with Fibrosis (n = 16)	p-value
**Anthropometric and clinical parameters** (normal distribution of data)				
Age (years)	42 ± 10	44 ± 10	42 ± 12	ns
Gender (% Male)	12%	54%	44%	**p<0.001** *
Weight (kg)	116 ± 17	125 ± 18	130 ± 30	ns
BMI (kg/cm^2^)	42 ± 5	42 ± 6	44 ± 11	ns
Body fat percentage (%)	54 ± 5	47 ± 6	49 ± 9	**p<0.001**
Waist circumference (cm)	120 ± 12	129 ± 10	127 ± 12	**p = 0.008**
Waist-to-hip ratio	0.95 ± 0.09	1.0 ± 0.09	1.04 ± 0.14	**p<0.001**
Smoking status(% smokers per group)	26%	27%	31%	ns*
**Biochemical parameters** (data not-normally distributed)				
ALT (U/L)	24[22–34]	30[22–39]	52[25–88]	**p = 0.03**
AST (U/L)	21 [20–28]	33[25–51]	40[26–88]	**p<0.001**
ALP (U/L)	87[73–106]	77[69–95]	81[69–113]	ns
GGT (U/L)	31[22–49]	39[34–48]	37[28–55]	ns
Ferritin (ng/ml)	62[28–95]	134[57–217]	119[53–335]	**p = 0.001**
Total cholesterol (mmol/L)	5.7[4.8–6.1]	5.1 [4.5–5.6]	4.9[4.1–5.7]	ns
LDL (mmol/L)	3.3 [2.7–3.8]	3.1 [2.6–3.7]	3.2 [2.4–3.7]	ns
HDL (mmol/L)	1.4 [1.1–1.7]	1.1[0.9–1.3]	1.0[0.9–1.2]	**p<0.001**
Triglycerides (mmol/L)	1.4[1.1–1.6]	1.7 [1.3–2.1]	1.5[1.1–2.7]	Ns
Fasting glucose (mmol/L)	4.3[4.1–5.0]	4.7[4.4–5.3]	5.0[4.4–6.1]	**p = 0.02**
Fasting insulin (μUnits/L)	15[10–18]	22[16–32]	25[17–32]	**p<0.001**
HOMA-IR	2.8[1.9–3.7]	4.4[3.3–7.0]	5.4[4.2–9.0]	**p<0.001**
C-Reactive Protein (nmol/L)	0.7[0.5–1.5]	0.5[0.3–1.3]	0.9[0.3–1.6]	ns
White cell count (x10^9^/L)	8.0[7.2–9.7]	8.2[6.6–9.0]	7.8[5.5–10]	ns
**Biomarkers, Cytokines and Chemokines**				
LPS (EU/ml)	2.6[2.3–2.9]	2.2[1.9–2.8]	2.8[2.4–3.0]	ns
LBP (ug/ml)	14[10–18]	15[9–24]	13[11–23]	ns
iFABP (pg/ml)	211[106–303]	238[125–383]	219[138–379]	ns
sCD14 (μg/ml)	2.4[2.1–3.1]	2.4[2.0–2.6]	2.7[2.3–3.1]	ns
CCL2 (pg/ml)	143[106–183]	118[77–169]	140[117–206]	ns
CCL3 (pg/ml)	6.7[5.9–8.0]	7.4[6.3–9.0]	9.3[7.5–11.6]	**p = 0.001**
IL6 (pg/ml)	0.6[0.4–0.8]	0.7[0.5–0.9]	0.7[0.5–1.2]	ns
IL8 (pg/ml)	1.7[1.2–2.4]	1.8[1.3–2.5]	3.3[1.8–4.3]	**p = 0.03**
TNFα (pg/ml)	1.1[0.9–1.3]	1.3[1.1–1.6]	1.3[1.1–1.6]	**p = 0.004**
TLR2 (ng/ml)	2.1[1.5–3.1]	2.5[1.8–2.8]	1.8[1.5–2.9]	ns
TLR4 (ng/ml)	2.4 [1.9–3.9]	2.7[1.8–3.9]	2.3[0.9–4.2]	ns

Data are given as mean +/- SD when they were shown to have a normal distribution or as median with [IQR] when they had a not-normal distribution.

Kruskal-Wallis test or Wilcoxon Rank Sum test were used where appropriate to determine differences between groups, a p<0.05 was considered significant. *) for proportional data the Chi-squared test was used

#### Demographics and anthropometric measurements

There were no significant differences between the NAFLD sub-groups with regards to age, weight and body mass index (BMI). Waist circumference and waist-to-hip ratio (WHR) was significantly higher in patients with NASH compared to No NAFL or NAFL groups (Table 1).

#### Biochemical analysis

Transaminase, ferritin levels and homeostasis model of assessment insulin resistance (HOMA IR) scores were higher in patients with more advanced liver disease (NASH and NASH with fibrosis) compared to patients with NAFL (Table 1). High density lipoproteins (HDL) were lower in patients with NASH (Table 1). Alanine aminotransferase (ALT), aspartate aminotransferase (AST) and Ferritin correlated with NAFLD activity score (NAS) (r = 0.442, p <0.001 and r = 0.474, p <0.001 and r = 0.405, p<0.001 respectively). Ferritin also positively correlated with ALT (r = 0.442, p <0.001) and AST (r = 0.378, p<0.001).

#### Plasma LPS correlates with markers of the metabolic syndrome but not with liver inflammation

**Fig 2:**
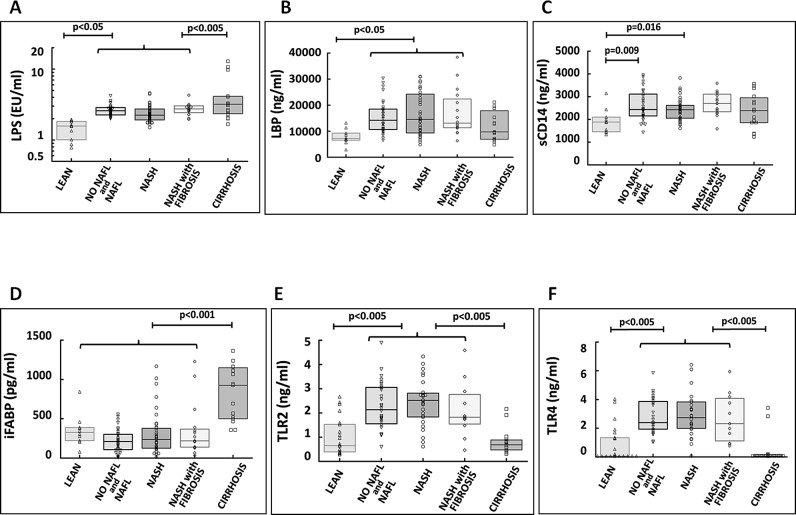
Plasma levels of markers of endotoxemia and intestinal permeability across the different histological subgroups of patients. Five out of six markers investigated (with exception of iFABP), the concentrations in the lean controls was significantly lower than in the NAFLD subgroups. For 4 markers (LPS, iFABP, TLR2 and TLR4) plasma levels in cirrhosis patients was significantly different from NAFLD subgroups. Brackets indicate that analysis of multiple subgroups with cirrhosis or lean group (as indicated) was statistically relevant for each subgroup separately. When comparison was made between the groups included in the brackets there was no statistical difference (Kruskal-Wallis One Way Analysis of Variance on Ranks). Only if p < 0.05, this was considered significant and indicated in the figures.

**Fig 3:**
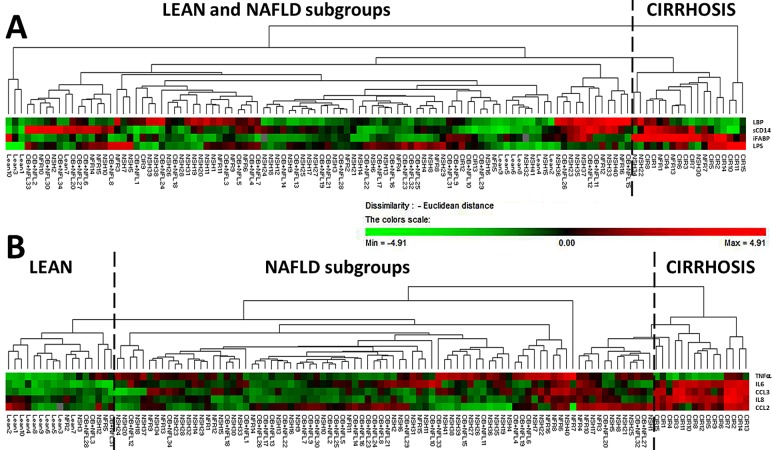
Hierarchic clustering was performed of patients using plasma levels of cytokines, chemokines and markers of endotoxemia to investigate global distribution along the 5 histological subgroups. **Fig 3A.** Hierarchic cluster analysis was performed using iFABP, LBP, LPS and sCD14. Using these markers only a group enriched with cirrhotic patients could be distinguished from the rest. In **Fig 3B** we used clustering on 5 cytokine/inflammation markers (CCL2, CCL3, TNFα, IL6 and IL8), whereby 3 blocks could be distinguished with some patients misclassified, using the same statistical setting (Euclidian distance, McQuitty’s linkage rule and normalized Z-score, see [22]). Abbreviations: CIR: cirrhosis; OB: obese with no NAFL; NFL: NAFL; NFR: NASH with fibrosis and NSH: NASH.

LPS forms complexes with lipopolysaccharide binding protein (LBP) and CD14 receptors [23]. Plasma LPS and LBP levels were significantly higher in the bariatric population compared to lean controls (Table 1 and S4 Table). However, plasma LPS and LBP levels were not different between the NAFLD subgroups and did not differ in obese individuals with normal liver histology compared with patients with NASH (Table 1 and Fig 2A and 2B). LPS levels correlated with body fat percentage (r = 0.209, p = 0.04) and C-reactive protein (r = 0.513, p<0.001) confirming its association with markers of the metabolic syndrome and inflammation. LPS and LBP levels did not correlate with plasma AST levels, liver inflammation, NAS or fibrosis scores nor did they correlate with waist-to-hip ratio (S5 Table). LPS levels weakly correlated with ALT levels (r = 0.217, p = 0.04). Plasma LPS levels were not different between current and previous smokers (S1 Fig). Plasma LPS levels positively correlated with plasma IL6, CCL2 and CCL3 (r = 0.399, p<0.001 and r = 0.371, p<0.001 and r = 0.358, p<0.001 respectively) (S2 Fig). These cytokines/chemokines increased especially during the development of cirrhosis suggesting that LPS may associate with the release of these proinflammatory cytokines/chemokines as the intestinal barrier fails with disease progression. To evaluate overall changes in plasma levels of cytokines, chemokines and markers of endotoxemia and to investigate whether these markers could be used to discriminate between the histological subgroups we performed hierarchic clustering analysis. Hierarchic cluster analysis using LPS-related markers (LPS, LBP, iFABP and sCD14) failed to discriminate between lean controls, NAFLD subgroups and only identified the NASH cirrhotics group (Fig 3A). Using the same strategy incorporating 5 cytokine/inflammation markers (CCL2, CCL3, TNFα, IL6 and IL8) hierarchic cluster analysis could distinguish lean, NAFLD subgroups and decompensated NASH cirrhotics although more patients were misclassified (Fig 3B).

#### Plasma sCD14 and iFABP are not elevated across the histological subgroups of NAFLD

sCD14 levels increase in response to bacterial infections and endotoxin exposure [24]. iFABP is a marker of intestinal epithelial dysfunction and increases if the epithelial barrier is compromised or injured [25]. Plasma sCD14 and iFABP levels were not significantly different between the subgroups (Table 1 and Fig 2C and 2D). iFABP levels increased significantly in NASH with cirrhosis. We also found a significant correlation between plasma iFABP and IL8 as well as iFABP and CCL3 levels (r = 309, p<0.001 and r = 0.229, p = 0.01 respectively) suggesting that increased epithelial dysfunction is associated with higher levels of these inflammatory cytokines/chemokines.

#### Plasma Toll-like receptors were significantly increased in the obese bariatric population compared to lean controls

Plasma TLR2 and TLR4 levels were significantly higher in the NAFLD subgroups compared to lean controls similar to what has been observed for LPS and LBP. TLR2 and TLR4 levels were however not different between the NAFLD subgroups (Fig 2E and 2F). We noted significantly lower levels of TLR2, 4 in cirrhosis most likely associated with cirrhosis associated immune dysfunction.

#### Inflammatory cytokines and chemokines correlate with liver inflammation and fibrosis

**Fig 4:**
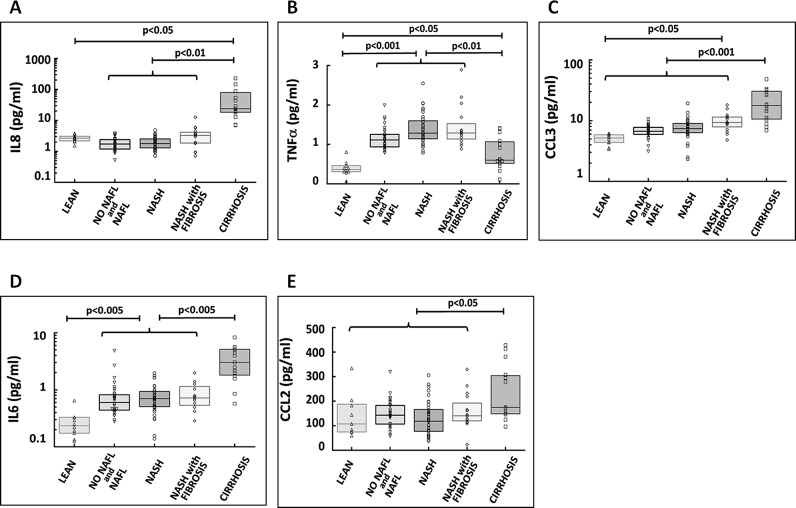
Plasma cytokine and chemokine levels across histological subgroups. Plasma IL8, TNFα and CCL3 were significantly different between histological subgroups (Fig 4A-4C). Plasma IL6 and CCL2 were significantly higher in patients with cirrhosis compared to other patient groups (Fig 4D and 4E) (see also Fig 2).

**Fig 5:**
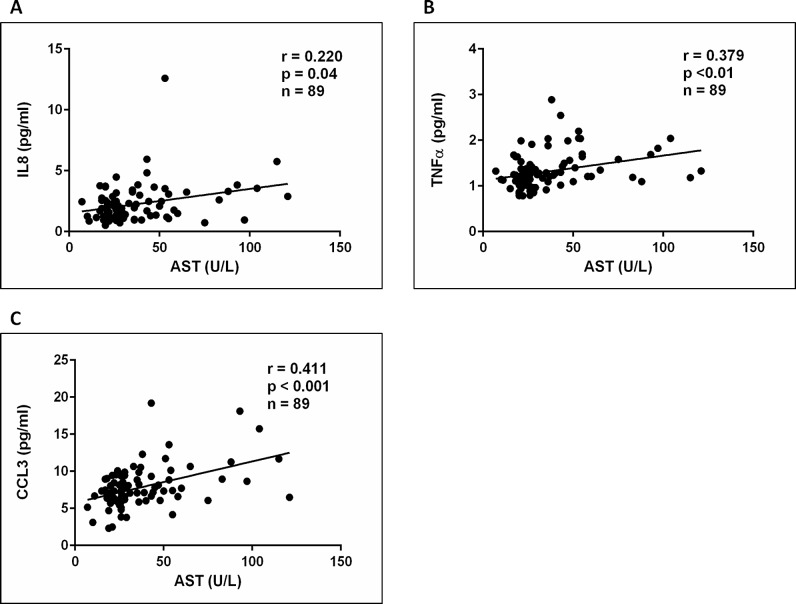
Pro-inflammatory cytokines correlate AST levels. Using Spearman correlation analysis we detected a significantly, positive correlation between plasma AST levels and cytokines IL8, TNFα and the chemokine CCL3 (Fig 5A-5C).

**Fig 6:**
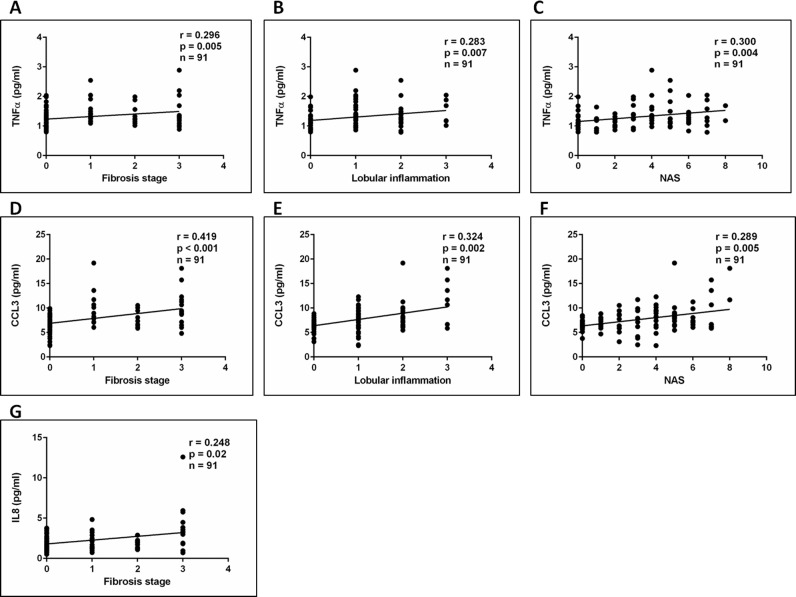
Spearman correlation between plasma levels of inflammatory cytokines and liver histology. Plasma TNFα and CCL3 correlate significantly with features of liver histology (Fig 6A-6F), such as NAS, lobular inflammation and fibrosis scores, while plasma IL8 levels correlated with fibrosis stage (Fig 6G) suggesting that these inflammatory mediators might be important in NAFLD pathogenesis.

Significant differences were detected in the plasma levels of the pro-inflammatory cytokines IL8, TNFα and CCL3 between the histological subgroups (Fig 4A–4C). TNFα and CCL3 correlated with waist-to-hip ratio but not with body fat percentage. In addition, TNFα and CCL3 also correlated with fasting insulin while a strong positive correlation was found between TNFα, CCL3 and HOMA-IR (S3 Fig). A significant correlation was also detected between IL8, TNFα, CCL3 and AST levels (Fig 5A–5C). Furthermore, TNFα and CCL3 levels positively correlated with NAS score, lobular inflammation and fibrosis score (Fig 6A–6F). IL8 correlated with fibrosis score (Fig 6G). AST has previously been shown to correlate with liver fibrosis suggesting that the cytokines/chemokines studied may be important mediators of the development of fibrosis in NASH [26]. Of note plasma IL8 and CCL3 levels were significantly elevated in patients with NASH and fibrosis compared to the other patient groups (Table 1). Plasma IL6 and CCL2 were significantly increased in the bariatric population compared to lean controls, while high levels were detected in patients with cirrhosis which is in keeping with previous publications from our group [27]. We also established a significant correlation between IL6 levels, waist circumference and HOMA-IR (S4 Fig).

## Discussion

The pathogenesis of NASH is unknown but is characterized by an increased delivery of lipotoxic free fatty acids (FFA’s) to the liver which is associated with persistent low grade systemic inflammation [28]. LPS levels are elevated in animal models of obesity and steatosis and are also increased in obese patients with NASH when compared to a healthy control population [29,30]. These findings have suggested that LPS translocation may be the main promoter of NASH progression in humans. However, in stark contrast to mouse models where steatohepatitis is universal and eventually develops in all animals on a high fat diet, NASH only develops in 20% of obese individuals suggesting that the pathogenesis of NAFLD may be different and more complex in human beings.

In this study we hypothesized that if LPS drives the development of NASH that LPS levels would be higher in individuals with NASH compared to NAFL, and would correlate with the histological severity of NAFLD. We used a novel approach by comprehensively assessing LPS and associated biomarkers, iFABP a marker of intestinal integrity, as well as pro-inflammatory cytokine levels in a well-matched bariatric patient population stratified into subgroups that reflected different stages of NAFLD progression. This approach allowed us to examine differences between markers of endotoxemia and pro-inflammatory cytokines in obese patients with normal liver histology or NAFL compared to individuals with NASH that were otherwise similar with regards to age and BMI.

LPS found on the outer wall of gram negative bacteria may cross the mucosal barrier in health and disease and is neutralized by circulating immunoglobulins and LBP [23]. LPS may form complexes with myeloid differentiation-2 (MD-2)/Toll-like receptor 4 (TLR4) which in turn activates nuclear factor κβ (NFκβ), inducing inflammatory cytokine production, and the release of sCD14 by myeloid cells [25]. Increased LPS levels in murine models have been clearly associated with systemic inflammation that induced obesity, insulin resistance and steatosis referred to as “metabolic endotoxaemia” even in the absence of a high fat diet [8]. In our study we specifically addressed whether i) LPS levels are different when comparing obese patients with various degrees of NALFD and if ii) LPS levels correlated with the histological severity of NAFLD in obese bariatric patients.

Our study showed that LPS, LBP and sCD14 levels were increased in all NAFLD subgroups compared to healthy blood donor controls. This finding again confirmed, as has been previously reported in literature, that parameters of endotoxaemia are elevated in obese individuals [24,31,32]. However, when we compared LPS levels (as well as LBP and sCD14 levels) between bariatric patients with limited liver disease to patients with NASH/NASH and fibrosis we found that LPS levels were similar between the subgroups. LPS levels however significantly increased in our cohort of decompensated NASH cirrhotics confirming that LPS translocation is especially pronounced when NASH patients develop decompensated cirrhosis. Of specific importance neither LPS, LBP or sCD14 correlated with AST levels, the histological severity of NAFLD, and fibrosis scores as reflected by the NAFLD Activity Score (NAS). Hierarchic clustering using markers of endotoxemia to investigate global distribution along the histological subgroups also failed to discriminate between the NAFLD subgroups suggesting that LPS is not the main factor associated with NAFL to NASH progression. The association of LPS with obesity and inflammation was highlighted by the positive correlation with body fat percentage and high CRP levels. In addition, LPS levels also correlated with the pro-inflammatory cytokine IL6, and the chemokines CCL2 and CCL3 suggesting that LPS is associated with systemic inflammation and monocyte recruitment especially when cirrhosis develops, a results which is in keeping with previous findings from our laboratory [27].

Pointing towards the presence of a low grade inflammatory state, we detected increased levels of TNFα, CCL3 and IL8 in NASH patients. Importantly these cytokines /chemokines correlated with transaminase levels and with the severity of the steatohepatitis suggesting that they may be important mediators in NAFLD-to-NASH progression. Recently we showed that CCR2+ macrophages isolated from adipose tissue at the time of bariatric surgery secrete pro-inflammatory cytokines/chemokines including TNFα, IL8, IL1β and CCL3 in bariatric patients with NASH. This suggested that this site may be a dominant source from where these cytokines are released [19].

Inflammatory cytokines have been extensively studied in both murine models and in patients with NASH. TNFα levels are increased in adult and paediatric NASH subjects compared to controls [33,34]. Ob/ob mice display several immunological abnormalities, including increased production of TNFα by inflammatory cells. Recently Engstler and coworkers demonstrated that TNFα inhibits Alcohol dehydrogenase (ADH) activity in Ob/Ob mice leading to higher ethanol serum levels in these animals. These findings linked elevated TNFα levels to the development of insulin resistance and the development of NAFLD [33]. Similar to the findings in mice we could also demonstrate that TNFα and CCL3 levels correlated with fasting glucose HOMA-IR and NAFLD severity suggesting that similar pathogenic mechanisms may underlie the development of insulin resistance and NASH in humans. IL8 is a potent leukocyte chemotactic chemokine and is secreted by various cells including monocytes and macrophages. Elevated IL8 levels have been observed in patients with chronic liver disease, and may contribute to hepatic inflammation by activating Kupffer cells. Adipose tissue of patients with NASH secrete more IL8 and elevated IL8 serum levels have been detected in NASH cirrhotics [19]. In obese Hispanic pediatric patients serum IL8 correlated with the hepatic fat fraction measured by MRI [35]. CCL3 has been shown to be a mediator of experimental liver fibrosis in mice [36].

The bacterial flora is altered in obesity and it has recently emerged that Myeloid differentiation primary response gene 88 (MyD88) the adaptor molecule central to all Toll-like receptors (TLRs) is crucial in initiating immune responses to altered bacterial flora [37]. We demonstrated that TLR2 and TLR4 were elevated in all bariatric subgroups compared to blood donor controls that mirrored the changes observed for LPS. This suggests that altered intestinal flora, associated with obesity, may activate downstream inflammatory pathways through MyD88 leading to increased systemic TLR2 and 4 levels and a pro-inflammatory environment.

The role of LPS in the development of NASH remains controversial. Some studies have found increased antibodies to LPS [32], higher LBP [31] and sCD14 levels [24] while others studies found no difference in LPS levels in NASH patients [34,38]. A recent study in NAFLD patients showed that gut permeability and the prevalence of small intestinal bacterial overgrowth correlated with liver steatosis but not with the presence of NASH. This suggests that factors other than bacterial flora are necessary to promote liver inflammation beyond the development of steatosis [39]. Our study supports this observation: elevated levels of LPS occurred across the spectrum of NAFLD suggesting that altered bacterial flora and elevated LPS levels may initiate inflammation but that other factors are necessary in progression to NASH. Rather than a single dominant pathway mediated through LPS binding to TLR4, various pathways independent of LPS exist that may induce a chronic systemic inflammatory state and steatohepatitis. In fact, recent work suggests that mitochondrial DNA released in microparticles derived from hepatocytes may activate TLR9 that may induce macrophages activation and upregulate NFκB–dependent proinflammatory cytokines production [40].

Recently Zhu and colleagues suggested a novel mechanism by which altered bacterial flora in NASH individuals may be linked to liver inflammation. They could demonstrate that gut microbiota enriched in alcohol-producing bacteria produced more alcohol than healthy microbiota. In that study, conducted in an obese pediatric population, they demonstrated that elevated alcohol levels could be observed in patients with NASH but not in obese patients without NASH [18].

In conclusion our study demonstrated that LPS and associated biomarkers, as well as TLR2, and TLR4 levels were elevated in obese bariatric patients compared to healthy controls. However, the levels of these parameters were not different in a large cohort of well-matched bariatric patients with different histological grades/stages of NAFLD. Instead we showed an association between elevated levels of circulating pro-inflammatory cytokines/chemokines in NAFL-to-NASH progression. The development of NASH in human beings is more complex than initially considered and not only dependent on elevated LPS levels.

## Supporting Information

S1 Table Detailed description of NAS (NAFLD activity score) histological scoring system. (DOCX)

S2 Table Lower limits of detection as specified by the manufacturer. (DOCX)

S3 Table Clinical and biochemical characteristics of the patient groups. (DOCX)

S4 Table Plasma levels of biomarkers and proinflammatory cytokines and chemokines measured across the five patient groups. (DOCX)

S5 Table Spearman correlation analysis between LPS, LBP levels and plasma AST levels or NAS. (DOCX)

S1 Fig Correlation analysis of LBP or LPS levels in the blood with smoking behavior. Patients were classified into 3 groups according their smoking behavior and LBP or LPS levels were graphically presented. No difference was detected in plasma LBP (Fig 1A) and LPS (Fig 1B) levels between current and previous smokers (0 = non-smoker, 1 = current smoker and 2 = former smoker (if stopped smoking >1 year)).
(DOCX)

S2 Fig Correlation of the pro-inflammatory mediators with plasma LPS levels. Plasma LPS significantly correlated with the cytokine IL6 (Fig 2A) and chemokines CCL2 and CCL3 (Fig 2B and 2C).
(DOCX)

S3 Fig Graphical presentation of the correlation of the pro-inflammatory mediators TNFα and CCL3 with waist-to-hip-ratio, fasting insulin and HOMA-IR in patients (NAFLD subgroups). A significant positively correlated was found between the anthropometric measurement waist-to-hip-ratio and both TNFα and CCL3 (Fig 3A and 3B). In addition, TNFα and CCL3 also correlated with fasting insulin and HOMA-IR (Fig 3C–3F).
(DOCX)

S4 Fig Correlation between plasma IL6 and waist circumference and HOMA-IR in NAFLD subgroups (Fig 4A and 4B). (DOCX)
