# Supplementary material for: Pro-Inflammatory Cytokines but Not Endotoxin-Related Parameters Associate with Disease Severity in Patients with NAFLD
Source: PLoS One. 2016 Dec 19;11(12):e0166048. doi: 10.1371/journal.pone.0166048 (PMC5167229; doi:10.1371/journal.pone.0166048)
Supplement: S1 Table — (DOCX) [file pone.0166048.s001.docx]

**S1:**

**Pro-inflammatory cytokines but not endotoxin-related parameters associate with disease severity in patients with NAFLD**

**Johannie du Plessis^1^**, **Hannelie Korf^1&2^, Jos van Pelt^1^, Petra Windmolders^1^**, **Ingrid Vander Elst^1^, An Verrijken^3^**, **Guy Hubens^4^**, **Luc Van Gaal^5^**, **David Cassiman^1,6^**, **Frederik Nevens^1,6^**, **Sven Francque^5^**, **Schalk van der Merwe^1,6^**

^1^Laboratory of Hepatology, KU Leuven, Leuven, Belgium

^2^Translational Research Center for Gastrointestinal Disorders (TARGID), Department of Clinical and Experimental Medicine, KU Leuven, Leuven, Belgium

^3^Department of Endocrinology, Diabetology and Metabolism, Antwerp University Hospital,

University of Antwerp, Antwerp, Belgium.

^4^Department of Abdominal Surgery, Antwerp University Hospital, University of Antwerp, Antwerp, Belgium

^5^Department of Gastroenterology and Hepatology, Antwerp University Hospital, University of Antwerp, Antwerp, Belgium.

^6^ Department of Internal Medicine, Division of Liver and biliopancreatic disorders, KU Leuven, Leuven, Belgium

**S1 Table: Detailed description of NAS (NAFLD activity score) histological scoring system**

| Fibrosis stage | 0 = none  1 = perisinusoidal or periportal  2 = perisinusoidal and portal/periportal  3 = bridging fibrosis  4 = cirrhosis |
| --- | --- |
| Steatosis grade | 0 = <5%  1 = 5%-33%  2 = >33%-66%  3 = >66% |
| Ballooning | 0 = none  1 = few balloon cells  2 = many cells/prominent ballooning |
| Lobular inflammation | 0 = no foci  1 = <2 foci per 200x field  2 = 2-4 foci per 200x field  3 = >4 foci per 200x field |
