# Supplementary material for: Pro-Inflammatory Cytokines but Not Endotoxin-Related Parameters Associate with Disease Severity in Patients with NAFLD
Source: PLoS One. 2016 Dec 19;11(12):e0166048. doi: 10.1371/journal.pone.0166048 (PMC5167229; doi:10.1371/journal.pone.0166048)
Supplement: S2 Table — (DOCX) [file pone.0166048.s002.docx]

**S2:**

**Pro-inflammatory cytokines but not endotoxin-related parameters associate with disease severity in patients with NAFLD**

**Johannie du Plessis^1^**, **Hannelie Korf^1&2^, Jos van Pelt^1^, Petra Windmolders^1^**, **Ingrid Vander Elst^1^, An Verrijken^3^**, **Guy Hubens^4^**, **Luc Van Gaal^5^**, **David Cassiman^1,6^**, **Frederik Nevens^1,6^**, **Sven Francque^5^**, **Schalk van der Merwe^1,6^**

^1^Laboratory of Hepatology, KU Leuven, Leuven, Belgium

^2^Translational Research Center for Gastrointestinal Disorders (TARGID), Department of Clinical and Experimental Medicine, KU Leuven, Leuven, Belgium

^3^Department of Endocrinology, Diabetology and Metabolism, Antwerp University Hospital,

University of Antwerp, Antwerp, Belgium.

^4^Department of Abdominal Surgery, Antwerp University Hospital, University of Antwerp, Antwerp, Belgium

^5^Department of Gastroenterology and Hepatology, Antwerp University Hospital, University of Antwerp, Antwerp, Belgium.

^6^ Department of Internal Medicine, Division of Liver and biliopancreatic disorders, KU Leuven, Leuven, Belgium

**S2 Table: Lower limits of detection as specified by the manufacturer**

| Protein measured | Lower limit of detection | Assay |
| --- | --- | --- |
| LPS | 0.1 EU/ml | Limulus Amoebocyte Lysate (LAL) assay QCL-1000 (Lonza) |
| LBP | 4.4 ng/ml | HyCult ELISA |
| sCD14 | 1.56 ng/ml | HyCult ELISA |
| iFABP | 47 pg/ml | HyCult ELISA |
| TLR2 | 0.119ng/ml | ELISA, Cloud-Clone Corporation |
| TLR4 | 0.060ng/ml | ELISA, Cloud-Clone Corporation |
| TLR9 | 0.054ng/ml | ELISA,Cloud-Clone Corporation |
| IL10 | 0.03 pg/ml | MSD V-PLEX |
| IL1β | 0.04 pg/ml | MSD V-PLEX |
| IL6 | 0.06 pg/ml | MSD V-PLEX |
| IL8 | 0.08 pg/ml | MSD V-PLEX |
| TNFα | 0.04 pg/ml | MSD V-PLEX |
| MCP1 | 0.09 pg/ml | MSD V-PLEX |
| MCP4 | 1.69 pg/ml | MSD V-PLEX |
| MDC | 1.22 pg/ml | MSD V-PLEX |
| MIP1α | 3.02 pg/ml | MSD V-PLEX |
