# Supplementary material for: Pro-Inflammatory Cytokines but Not Endotoxin-Related Parameters Associate with Disease Severity in Patients with NAFLD
Source: PLoS One. 2016 Dec 19;11(12):e0166048. doi: 10.1371/journal.pone.0166048 (PMC5167229; doi:10.1371/journal.pone.0166048)
Supplement: S3 Table — (DOCX) [file pone.0166048.s003.docx]

**S3:**

**Pro-inflammatory cytokines but not endotoxin-related parameters associate with disease severity in patients with NAFLD**

**Johannie du Plessis^1^**, **Hannelie Korf^1&2^, Jos van Pelt^1^, Petra Windmolders^1^**, **Ingrid Vander Elst^1^, An Verrijken^3^**, **Guy Hubens^4^**, **Luc Van Gaal^5^**, **David Cassiman^1,6^**, **Frederik Nevens^1,6^**, **Sven Francque^5^**, **Schalk van der Merwe^1,6^**

^1^Laboratory of Hepatology, KU Leuven, Leuven, Belgium

^2^Translational Research Center for Gastrointestinal Disorders (TARGID), Department of Clinical and Experimental Medicine, KU Leuven, Leuven, Belgium

^3^Department of Endocrinology, Diabetology and Metabolism, Antwerp University Hospital,

University of Antwerp, Antwerp, Belgium.

^4^Department of Abdominal Surgery, Antwerp University Hospital, University of Antwerp, Antwerp, Belgium

^5^Department of Gastroenterology and Hepatology, Antwerp University Hospital, University of Antwerp, Antwerp, Belgium.

^6^ Department of Internal Medicine, Division of Liver and biliopancreatic disorders, KU Leuven, Leuven, Belgium

**S3 Table: Clinical and biochemical characteristics of the patient groups**

|  | **LEAN** | **No NAFL and NAFL** | **NASH** | **NASH with Fibrosis**  **(n=16)** | **Cirrhosis** | **p-value** |
| --- | --- | --- | --- | --- | --- | --- |
|  | **(n=10)** | **(n=34)** | **(n=41)** |  | **(n=15)** |  |
| **Anthropometric and Clinical parameters** | | | | | | |
| Age (years) | ** | 42 ± 10 | 44 ± 10 | 42 ± 12 | 59± 11 | **0.002** |
| Gender (% Male) | ** | 12% | 54% | 44% | 56% | **<0.001*** |
| **Biochemical parameters** | | | | | | |
| ALT (U/L) | 11[9-14] | 24[22-34] | 30[22-39] | 52[25-88] | 39[20-50] | **0.05** |
| AST (U/L) | 25[21-31] | 21 [20-28] | 33[25-51] | 40[26-88] | 54 [35-68] | **<0.001** |
| ALP (U/L) | n.d. | 87[73-106] | 77[69-95] | 81[69-113] | 99 [71-124] | **ns** |
| GGT (U/L) | 17[13-19] | 31[22-49] | 39[34-48] | 37[28-55] | 49 [15-138] | **ns** |
| Ferritin (ng/ml) | 94[82-152] | 62[28-95] | 134[57-217] | 119[53-335] | 289[76-691] | **0.001** |
| Total cholesterol (mmol/L) | 4.1[4.0-5.3] | 5.7[4.8-6.1] | 5.1 [4.5-5.6] | 4.9[4.1-5.7] | 4.0[2.0-4.6] | **0.001** |
| Triglycerides (mmol/L) | 0.8[0.7-1.3] | 1.4[1.1-1.6] | 1.7[ 1.3-2.1] | 1.5[1.1-2.7] | 0.84[0.53-1.08] | **0.002** |
| Fasting glucose (mmol/L) | n.d. | 4.3[4.1-5.0] | 4.7[4.4-5.3] | 5.0[4.4-6.1] | 6.1[5.1-7.7] | **<0.001** |
| C-Reactive Protein (nmol/L) | 0.3[0.3-1] | 0.7[0.5-1.5] | 0.5[0.3-1.3] | 0.9[0.3-1.6] | 7.6[3.9-13.2] | **<0.001** |
| White cell count (x10^9^/L) | n.d. | 8.0[7.2-9.7] | 8.2[6.6-9.0] | 7.8[5.5-10] | 6.1[2.6-7.8] | **ns** |
| Data are given as mean +/- SD when they were shown to have a normal distribution or in case of biochemical parameters, that had a not-normal distribution, as median with [IQR]. | | | | | | |
| Kruskal-Wallis test or Wilcoxon Rank Sum test were used where appropriate to determine differences between groups, a p<0.05 was considered significant. *) for proportional data the Chi-squared test was used. ns=not significant  **) blood samples of adult lean controls were anonymous collected prior to biochemical analysis | | | | | | |
