# Supplementary material for: Pro-Inflammatory Cytokines but Not Endotoxin-Related Parameters Associate with Disease Severity in Patients with NAFLD
Source: PLoS One. 2016 Dec 19;11(12):e0166048. doi: 10.1371/journal.pone.0166048 (PMC5167229; doi:10.1371/journal.pone.0166048)
Supplement: S4 Table — (DOCX) [file pone.0166048.s004.docx]

**S4:**

**Pro-inflammatory cytokines but not endotoxin-related parameters associate with disease severity in patients with NAFLD**

**Johannie du Plessis^1^**, **Hannelie Korf^1&2^, Jos van Pelt^1^, Petra Windmolders^1^**, **Ingrid Vander Elst^1^, An Verrijken^3^**, **Guy Hubens^4^**, **Luc Van Gaal^5^**, **David Cassiman^1,6^**, **Frederik Nevens^1,6^**, **Sven Francque^5^**, **Schalk van der Merwe^1,6^**

^1^Laboratory of Hepatology, KU Leuven, Leuven, Belgium

^2^Translational Research Center for Gastrointestinal Disorders (TARGID), Department of Clinical and Experimental Medicine, KU Leuven, Leuven, Belgium

^3^Department of Endocrinology, Diabetology and Metabolism, Antwerp University Hospital,

University of Antwerp, Antwerp, Belgium.

^4^Department of Abdominal Surgery, Antwerp University Hospital, University of Antwerp, Antwerp, Belgium

^5^Department of Gastroenterology and Hepatology, Antwerp University Hospital, University of Antwerp, Antwerp, Belgium.

^6^ Department of Internal Medicine, Division of Liver and biliopancreatic disorders, KU Leuven, Leuven, Belgium

**S4 Table: Plasma levels of biomarkers and proinflammatory cytokines and chemokines measured across the five patient groups**

| **Biomarker** | **LEAN** | **No NAFL and NAFL** | **NASH** | **NASH with FIBROSIS** | **CIRRHOSIS** | **p-value** |
| --- | --- | --- | --- | --- | --- | --- |
|  | **(n=10)** | **(n=34)** | **(n=41)** | **(n=16)** | **(n=15)** |  |
| **LPS** | 1.6[1.0-1.8] | 2.6[2.3-2.9] | 2.2[1.9-2.8] | 2.8[2.4-3.0] | 3.2[2.3-4.1] | **<0.001** |
| **(EU/ml)** |  |  |  |  |  |  |
| **LBP** | 7.2[6.3-9.8] | 14.2[10-18] | 14.5[9.2-24] | 13.0[11-23] | 9.7[6.8-19] | **=0.002** |
| **(ug/ml)** |  |  |  |  |  |  |
| **IFABP** | 326[218-406] | 211[106-303] | 238[125-383] | 219[138-379] | 925[496-1160] | **<0.001** |
| **(pg/ml)** |  |  |  |  |  |  |
| **sCD14** | 1.9[1.4-2.2] | 2.4[2.1-3.1] | 2.4[2.0-2.6] | 2.7[2.3-3.1] | 2.4[1.9-3.0] | **<0.001** |
| **(µg/ml)** |  |  |  |  |  |  |
| **CCL2** | 107[74-193] | 143[106-183] | 118[77-169] | 140[117-206] | 175[147-306] | **=0.004** |
| **(pg/ml)** |  |  |  |  |  |  |
| **CCL3** | 5.2[4.2-5.9] | 6.7[5.9-8.0] | 7.4[6.3-9.0] | 9.3[7.5-11.6] | 18[10-31] | **<0.001** |
| **(pg/ml)** |  |  |  |  |  |  |
| **IL6** | 0.2[0.2-0.3] | 0.6[0.4-0.8] | 0.7[0.5-0.9] | 0.7[0.5-1.2] | 3 [1.7-5.2] | **<0.001** |
| **(pg/ml)** |  |  |  |  |  |  |
| **IL8** | 2.7[2.2-3.2] | 1.7[1.2-2.4] | 1.8[1.3-2.5] | 3.3[1.8-4.3] | 24[18-87] | **<0.001** |
| **(pg/ml)** |  |  |  |  |  |  |
| **TNFα** | 0.4[0.3-0.5] | 1.1[0.9-1.3] | 1.3[1.1-1.6] | 1.3[1.1-1.6] | 0.6[0.5-1.1] | **<0.001** |
| **(pg/ml)** |  |  |  |  |  |  |
| **TLR2** | 2.1[1.1-2.4] | 2.1[1.5-3.1] | 2.5[1.8-2.8] | 1.8[1.5-2.9] | 0.7[0.5-0.9] | **<0.001** |
| **(ng/ml)** |  |  |  |  |  |  |
| **TLR4** | 1.7[1.3-3.8] | 2.4 [1.9-3.9] | 2.7[2.0-3.9] | 2.3[0.9-4.2] | 0.3[0.2-0.3] | **=0.005** |
| **(ng/ml)** |  |  |  |  |  |  |
| Data are represented as median [IQR] | | | |  |  |  |
| Kruskal-Wallis test or Wilcoxon Rank Sum test was used to determine differences between groups, a p<0.05 was considered significant | | | | | | |
