# Supplementary material for: Pro-Inflammatory Cytokines but Not Endotoxin-Related Parameters Associate with Disease Severity in Patients with NAFLD
Source: PLoS One. 2016 Dec 19;11(12):e0166048. doi: 10.1371/journal.pone.0166048 (PMC5167229; doi:10.1371/journal.pone.0166048)
Supplement: S5 Table — (DOCX) [file pone.0166048.s005.docx]

**S5:**

**Pro-inflammatory cytokines but not endotoxin-related parameters associate with disease severity in patients with NAFLD**

**Johannie du Plessis^1^**, **Hannelie Korf^1&2^, Jos van Pelt^1^, Petra Windmolders^1^**, **Ingrid Vander Elst^1^, An Verrijken^3^**, **Guy Hubens^4^**, **Luc Van Gaal^5^**, **David Cassiman^1,6^**, **Frederik Nevens^1,6^**, **Sven Francque^5^**, **Schalk van der Merwe^1,6^**

^1^Laboratory of Hepatology, KU Leuven, Leuven, Belgium

^2^Translational Research Center for Gastrointestinal Disorders (TARGID), Department of Clinical and Experimental Medicine, KU Leuven, Leuven, Belgium

^3^Department of Endocrinology, Diabetology and Metabolism, Antwerp University Hospital,

University of Antwerp, Antwerp, Belgium.

^4^Department of Abdominal Surgery, Antwerp University Hospital, University of Antwerp, Antwerp, Belgium

^5^Department of Gastroenterology and Hepatology, Antwerp University Hospital, University of Antwerp, Antwerp, Belgium.

^6^ Department of Internal Medicine, Division of Liver and biliopancreatic disorders, KU Leuven, Leuven, Belgium

**S5 Table: Spearman correlation analysis between LPS, LBP levels and plasma AST levels or NAS**

|  | Plasma AST levels | | NAS | |
| --- | --- | --- | --- | --- |
| Biomarker | r-value | p-value | r-value | p-value |
| LPS | r = -0.156 | p = 0.1 | r = 0.01 | p = 0.9 |
| LBP | r = 0.120 | p = 0.2 | r= - 0.08 | p = 0.4 |
