# Supplementary material for: Pro-Inflammatory Cytokines but Not Endotoxin-Related Parameters Associate with Disease Severity in Patients with NAFLD
Source: PLoS One. 2016 Dec 19;11(12):e0166048. doi: 10.1371/journal.pone.0166048 (PMC5167229; doi:10.1371/journal.pone.0166048)
Supplement: S1 Fig — Patients were classified into 3 groups according their smoking behavior and LBP or LPS levels were graphically presented. No difference was detected in plasma LBP (Fig 1A) and LPS (Fig 1B) levels between current and previous smokers (0 = non-smoker, 1 = current smoker and 2 = former smoker (if stopped smoking >1 year)). (DOCX) [file pone.0166048.s006.docx]

**S1:**

**Pro-inflammatory cytokines but not endotoxin-related parameters associate with disease severity in patients with NAFLD**

**Johannie du Plessis^1^**, **Hannelie Korf^1&2^, Jos van Pelt^1^, Petra Windmolders^1^**, **Ingrid Vander Elst^1^, An Verrijken^3^**, **Guy Hubens^4^**, **Luc Van Gaal^5^**, **David Cassiman^1,6^**, **Frederik Nevens^1,6^**, **Sven Francque^5^**, **Schalk van der Merwe^1,6^**

^1^Laboratory of Hepatology, KU Leuven, Leuven, Belgium

^2^Translational Research Center for Gastrointestinal Disorders (TARGID), Department of Clinical and Experimental Medicine, KU Leuven, Leuven, Belgium

^3^Department of Endocrinology, Diabetology and Metabolism, Antwerp University Hospital,

University of Antwerp, Antwerp, Belgium.

^4^Department of Abdominal Surgery, Antwerp University Hospital, University of Antwerp, Antwerp, Belgium

^5^Department of Gastroenterology and Hepatology, Antwerp University Hospital, University of Antwerp, Antwerp, Belgium.

^6^ Department of Internal Medicine, Division of Liver and biliopancreatic disorders, KU Leuven, Leuven, Belgium







**B**

**A**

**S1 Fig: Correlation analysis of LBP or LPS levels in the blood with smoking behavior.** Patients were classified into 3 groups according their smoking behavior and LBP or LPS levels were graphically presented. No difference was detected in plasma LBP (Fig A) and LPS (Fig B) levels between current and previous smokers (0 = non-smoker, 1 = current smoker and 2 = former smoker (if stopped smoking >1 year)).
