# Supplementary material for: Pro-Inflammatory Cytokines but Not Endotoxin-Related Parameters Associate with Disease Severity in Patients with NAFLD
Source: PLoS One. 2016 Dec 19;11(12):e0166048. doi: 10.1371/journal.pone.0166048 (PMC5167229; doi:10.1371/journal.pone.0166048)
Supplement: S3 Fig — A significant positively correlated was found between the anthropometric measurement waist-to-hip-ratio and both TNFα and CCL3 (Fig 3A and 3B). In addition, TNFα and CCL3 also correlated with fasting insulin and HOMA-IR (Fig 3C–3F). (DOCX) [file pone.0166048.s008.docx]

**S3:**

**Pro-inflammatory cytokines but not endotoxin-related parameters associate with disease severity in patients with NAFLD**

**Johannie du Plessis^1^**, **Hannelie Korf^1&2^, Jos van Pelt^1^, Petra Windmolders^1^**, **Ingrid Vander Elst^1^, An Verrijken^3^**, **Guy Hubens^4^**, **Luc Van Gaal^5^**, **David Cassiman^1,6^**, **Frederik Nevens^1,6^**, **Sven Francque^5^**, **Schalk van der Merwe^1,6^**

^1^Laboratory of Hepatology, KU Leuven, Leuven, Belgium

^2^Translational Research Center for Gastrointestinal Disorders (TARGID), Department of Clinical and Experimental Medicine, KU Leuven, Leuven, Belgium

^3^Department of Endocrinology, Diabetology and Metabolism, Antwerp University Hospital,

University of Antwerp, Antwerp, Belgium.

^4^Department of Abdominal Surgery, Antwerp University Hospital, University of Antwerp, Antwerp, Belgium

^5^Department of Gastroenterology and Hepatology, Antwerp University Hospital, University of Antwerp, Antwerp, Belgium.

^6^ Department of Internal Medicine, Division of Liver and biliopancreatic disorders, KU Leuven, Leuven, Belgium















**D**

**F**

**E**

**C**

**B**

**A**

**S3 Fig: Graphical presentation of the correlation of the pro-inflammatory mediators TNFα and CCL3 with waist-to-hip-ratio, fasting insulin and HOMA-IR in patients (NAFLD subgroups).** A significant positively correlated was found between the anthropometric measurement waist-to-hip-ratio and both TNFα and CCL3 (Fig A and B). In addition, TNFα and CCL3 also correlated with fasting insulin and HOMA-IR (Fig C-F).
